# Risk factor analysis and nomogram prediction model construction for NEC complicated by intestinal perforation

**DOI:** 10.1186/s12887-024-04640-2

**Published:** 2024-02-27

**Authors:** Pei Huang, Nandu Luo, Xiaoqi Shi, Jiahong Yan, Jiaojiao Huang, Yan Chen, Zuochen Du

**Affiliations:** 1 Department of Pediatrics, Affiliated Hospital of Zunyi Medical University, Zunyi, China; 2 Department of Pediatrics, Guizhou Children’s Hospital, Zunyi, China; 3 Collaborative Innovation Center for Tissue Injury Repair and Regenerative Medicine of Zunyi Medical University, Zunyi, China

**Keywords:** Neonate, Necrotizing enterocolitis, Nomogram, Intestinal perforation, Risk factor

## Abstract

**Objective:**

To investigate the clinical characteristics of neonatal necrotizing enterocolitis (NEC) complicated by intestinal perforation and predict the incidence of intestinal perforation in NEC.

**Methods:**

Neonates diagnosed with NEC at the Affiliated Hospital of Zunyi Medical University from January 2012 to May 2022 were enrolled, and the clinical data were collected and analyzed retrospectively. The patients were divided into two groups based on intestinal perforation occurrence or not. Mann-Whitney U tests, t-tests, chi-square tests, and fisher’s exact tests were performed between-group comparisons. Logistic and lasso regressions were applied to screen independent risk factors for concomitant bowel perforation, and R software (RMS package) was used to formulate the nomogram prediction model. In addition, the receiver operating curve (ROC) and the calibration curve were drawn to verify the predictive power, while decision curve analysis (DCA) was constructed to evaluate the clinical applicability of the nomogram model.

**Results:**

One hundred eighty neonates with NEC were included, of which 48 had intestinal perforations, and 132 did not; the overall incidence of intestinal perforation was 26.67% (48/180). Bloody stool (OR = 5.60), APTT ≥ 50 s (OR = 3.22), thrombocytopenia (OR = 4.74), and hypoalbuminemia (OR = 5.56) were identified as independent risk variables for NEC intestinal perforation (*P* < 0.05) through multivariate logistic regression analysis. These factors were then applied to develop a nomogram prediction model (C-index = 0.838) by using the R software. The area under the curve (AUC) for the nomogram in the training and validation cohorts were 0.838 (95% Cl: 0.768, 0.908) and 0.802 (95% CI: 0.659, 0.944), respectively. The calibration curve shown that the nomogram has a good predictive ability for predicting the risk of intestinal perforation occurrence. And the decision curve and clinical impact curve analyses demonstrated good clinical utility of the nomogram model.

**Conclusion:**

We found that Bloody stool, APTT ≥ 50 s, Thrombocytopenia, and hypoalbuminemia could be used as independent risk factors for predicting intestinal perforation in neonates with NEC. The nomogram model based on these variables had high predictive values to identify NEC patients with intestinal perforation.

## Introduction

Neonatal necrotizing enterocolitis (NEC) is an acute necrotizing inflammatory disease involving the ileum and colon, with an incidence of (0.5–5)/1000 and a mortality rate of 10–20% [1, 2], which is one of the most common fatal diseases in the neonatal period [3, 4]. Neonates with necrotizing enterocolitis are susceptible to intestinal perforation, sepsis, coagulation malfunction, and multi-organ damage [5–7]. Intestinal perforation is one of the most serious complications [8]. Studies have indicated that the death rate of neonates with intestinal perforation is as high as 50% [9–12]. Therefore, early diagnosis of NEC in complications with intestinal perforation is crucial for the treatment of the disease.

As an important medical prediction tool [13], the nomogram can provide individual probabilities of clinical occurrences by integrating many prognostic risk factors, and is currently widely used for predicting the prognosis [13–16], recurrence [17, 18], and severity of many diseases [19, 20]. And it is a very accurate computerized model for clinical decision-making. However, there have been no reports of nomogram for predicting intestinal perforation in NEC.

The risk factors reported for NEC occurrence in infants are closely linked to maternal and prenatal factors (hypertension in pregnancy, convulsions, infections, etc.), infant factors (gestational age, delivery mode, birth weight, feeding pattern, etc.), and postnatal factors (sepsis, heart disease, septicemia, anemia, hypoxia-associated diseases, hypoproteinemia, blood transfusion, etc.) [21–23]. Also, a predictive score of NEC risk factors has been developed to find neonates at high risk [24]. The use of the nomogram prediction model to predict the risk of NEC and prognostic analysis of the different stages of NEC has been reported in the literature, which is helpful for clinical guidance [5]. However, although intestinal perforation is directly related to the prognosis of neonates with NEC, no relevant risk prediction model has been devised. In this study, the risk factors of NEC were collected to develop effective early predictors of intestinal perforation in NEC and to further construct a visualization scoring system for the independent risk variables to give a scientific and theoretical basis for reducing the morbidity and mortality rate of neonates with NEC.

## Materials and methods

### Patients

**Fig. 1:**
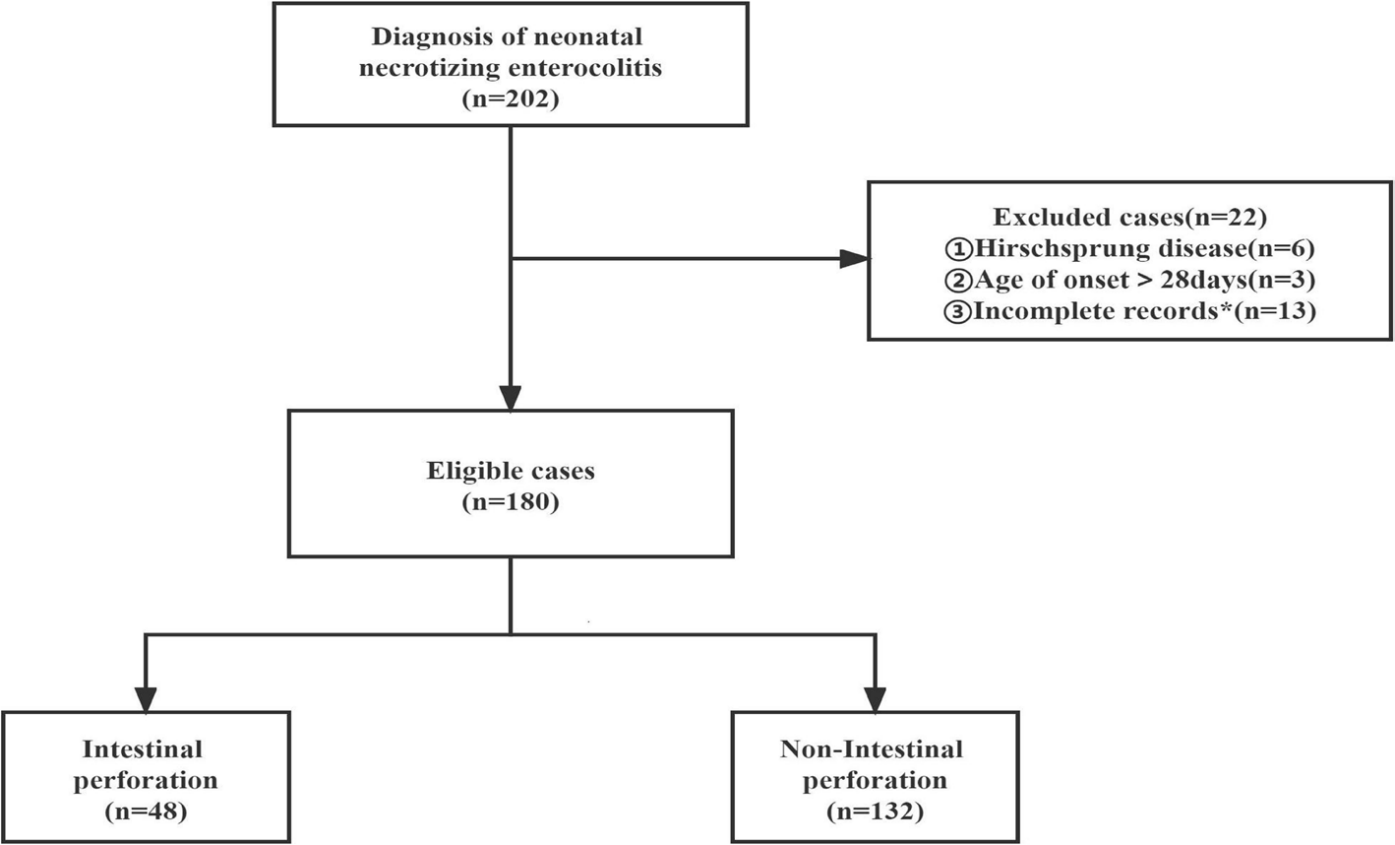
Flow chart for patient selection

Neonates diagnosed with NEC who were hospitalized in the Department of Pediatrics/Pediatrics Surgery of the Affiliated Hospital of Zunyi Medical University from January 2012 and May 2022 were enrolled. The patients were divided into two groups: perforated (48 cases) and non-perforated (132 cases), based on whether they had an intestinal perforation or not. Inclusion criteria for NEC were as follows: (a). Bell’s staging criteria were met [3]; (b). NEC was diagnosed during the newborn period (≤ 28 days); (c). Clinical data were complete. Exclusion criteria include: (a). Combined with congenital gastrointestinal abnormalities such as congenital megacolon, intestinal malrotation, and intestinal atresia; (b). Combined with severe congenital disabilities and hereditary metabolic illnesses; (c). Missing clinical data, as is showed in Fig. 1. Intestinal perforation diagnostic criteria are as follows: X-ray showed subdiaphragmatic free gas, encapsulated or confined pneumoperitoneum; laparotomy confirming the presence of GI contents in the abdominal cavity or perforation discovered during surgery [6, 25]. The study was approved by the Ethics Committee of the Affiliated Hospital of Zunyi Medical University (KLL-2022-657).

### Data collection

Demographic data, clinical information, and laboratory examinations were collected from the medical records of patients diagnosed with NEC(Collect these test and examination indicators within the first 5 days of NEC diagnosis), including gender, age at onset, birth weight, mode of delivery, premature birth status, absolute neutrophil count (ANC), platelet (PLT), lymphocyte (LYM), monocyte (MO), hemoglobin (HB), red blood cell distribution width (RDW), alanine aminotransferase (ALT), aspartate aminotransferase (AST), alkaline phosphatase(AKP), total bilirubin (TBIL), creatine kinase (CK), creatine kinase isoenzyme-MB(CK-MB), lactate dehydrogenase (LDH), prealbumin (PAB), creatinine (Cr), prothrombin time (PT), activated partial thromboplastin time (APTT). And the complications of NEC including pulmonary hemorrhage, thrombocytopenia (PLT < 100 × 10^9^/L), bloody stool, peritonitis, hypoproteinemia, pneumonia, respiratory failure, sepsis, metabolic acidosis, shock, neonatal hypoglycemia, disseminated intravascular coagulation (DIC), myocardial damage, coagulation abnormalities, electrolyte disturbances, sclerema neonatorum, cholestasis, hyperbilirubinemia were also collected.

### Statistical analysis

All data were processed and analyzed by using SPSS 26.0 and R language (4.2.1/RMS data package) software. The measurement data were described as mean ± standard deviation ($\overline{x }$± *s*), and the means were compared by unpaired t-test. Non-normal distributed data were described as median and quartiles (M (Q1, Q3)), and analyzed using Mann-Whitney U test. Count data were presented as n (%) and analyzed using chi-squared test.

Univariate analysis and LASSO regression were applied to screen the possible risk factors for concomitant bowel perforation of NEC, then multivariate logistic regression analysis was conducted based on the variables screened by LASSO regression. R software (RMS package) was used to formulate the nomogram prediction model. The receiver operating curve (ROC) and Harrell concordance index (C-index) analyses were performed for the validation and discrimination of the model. The calibration curves of the training and validation cohorts were plotted to evaluate the consistency between the predicted and observed probabilities. To assess the clinical applicability of the model, a decision curve analysis (DCA) was performed. All statistically significant tests were two-tailed, and *P* < 0.05 was regarded as statistically significant.

## Results

### The characteristics of neonatal necrotizing enterocolitis (NEC) complicated by intestinal perforation

This study eventually included 180 neonates, with 128 patients in the training cohort and 52 patients in the validation cohort using random split sampling in a 7:3 manner (Fig. 1). The demographic characteristics and general information of the included patients were summarized in Table 1. Most of the infants enrolled in this study were late preterm or term infants. 42.2% (76/180) of the infants were full-term infants, 26.1% (47/180) were mid-late preterm infants, and only 31.7% (57/180) had a gestational age of less than 32 weeks. Overall, 30.6% (55/180) of the infants had a low birth weight (≤ 1500 g), but the proportion of low birth weight (≤ 1500 g) patients in the perforation group (56.3%, 27/48) was significantly higher than that in the non-perforation group (21.2%,28/132). While there are no differences between the two groups or cohorts in terms of gender, age at onset, season, delivery method, or feeding method (*P* > 0.05) (Table 1).

**Table 1:** Demographics and general information of NEC patients

Characteristics	Training cohort（*n* = 128				Validation cohort（*n* = 52				*P#*
	Total (128)	Perforation (*n* = 34)	Non-Perforation (*n* = 94)	*P *value	Total (52)	Perforation (*n* = 14)	Non-Perforation (*n* = 38)	*P *value	
Gender	－	－	－	0.407	－	－	－	0.371	0.148
Male	79 (61.7)	23 (67.6)	56 (59.6)	－	38 (73.1)	12 (85.7)	26 (68.4)	－	－
Female	49 (38.3)	11 (32.4)	38 (40.4)	－	14 (26.9)	2 (14.3)	12 (31.6)	－	－
Age at onset(days)	－	－	－	0.113	－	－	－	0.588	0.488
≤ 14	92 (71.9)	28 (82.4)	64 (68.1)	－	40 (76.9)	12 (85.7)	28 (73.7)	－	－
> 14	36 (28.1)	6 (17.6)	30 (31.9)	－	12 (23.1)	2 (14.3)	10 (26.3)	－	－
Season	－	－	－	0.058	－	－	－	0.085	0.697
Spring	33 (25.8)	11 (32.4)	22 (23.4)	－	18 (34.6)	2 (14.3)	16 (42.1)	－	－
Summer	29 (22.7)	12 (35.3)	17 (18.1)	－	11 (21.2)	5 (35.7)	6 (15.8)	－	－
Autumn	38 (29.7)	7 (20.6)	31 (33.0)	－	13 (25.0)	4 (28.6)	9 (23.7)	－	－
Winter	28 (21.8)	4 (11.7)	24 (25.5)	－	10 (19.2)	3 (21.4)	7 (18.4)	－	－
Gestational age	－	－	－	0.502	－	－	－	0.118	0.378
≥ 37 weeks	51 (39.8)	11 (32.4)	40 (42.6)	－	25 (48.1)	4 (28.6)	21 (55.3)	－	－
32-37 weeks	37 (28.9)	10 (29.4)	27 (28.7)	－	10 (19.2)	5 (35.7)	5 (13.2)	－	－
≤ 32 weeks	40 (31.3)	13 (38.2)	27 (28.7)	－	17 (32.7)	5 (35.7)	12 (31.5)	－	－
Weight(g)	－	－	－	<0.001	－	－	－	0.949	0.014
> 1500	82 (64.1)	10 (29.4)	72 (76.6)	－	43 (82.7)	11 (78.6)	32 (84.2)	－	－
≤ 1500	46 (35.9)	24 (70.6)	22 (23.4)	－	9 (17.3)	3 (21.4)	6 (15.8)	－	－
Delivery way	－	－	－	0.262	－	－	－	0.532	0.775
Natural	61 (47.7)	19 (55.8)	42 (44.7)	－	26 (50.0)	8 (57.1)	18 (47.4)	－	－
Cesarean	67 (52.3)	15 (44.2)	52 (55.3)	－	26 (50.0)	6 (42.9)	20 (52.6)	－	－
Feeding way	－	－	－	0.442	－	－	－	1.000	0.059
Breast milk	21 (16.4)	7 (20.6)	14 (14.9)	－	15 (28.8)	4 (28.6)	11 (28.9)	－	－
Formula milk	107 (83.6)	27 (79.4)	80 (85.1)	－	37 (71.2)	10 (71.4)	27 (71.1)	－	－

#Comparison between the training and validation cohort variables

Looking at the clinical characteristics between the two groups in the training cohort, we found that bloody stool (47.1% *vs.* 16%, *P* < 0.001), peritonitis (41.2% *vs.* 17%, *P* = 0.004), thrombocytopenia (41.2% *vs.* 8.5%, *P* < 0.001), and hypoalbuminemia (52.9% *vs.* 19.1%, *P* < 0.001) were more common in the perforation group, while pneumonia, respiratory failure, pulmonary hemorrhage, metabolic acidosis, sepsis, anemia, electrolyte disorders, shock, and hypoglycemia showed no statistical significance (Table 2). A higher proportion of patients with peritonitis (50.0% *vs.* 10.5%, *P* = 0.004) or thrombocytopenia (35.7% *vs.* 5.3%, *P* < 0.001) was also detected in the validation cohort, while the other characteristics showed no significant difference between the two groups (Table 2).

**Table 2:** Clinical characteristics and laboratory findings of NEC patients

Characteristics	Training cohort (*n* = 128				Validation cohort (*n* = 52)				
	Total (*n* = 128）	Perforation (*n* = 34)	Non-Perforation (*n* = 94)	*P *value	Total (*n* = 52）	Perforation (*n* = 14)	Non-Perforation (*n* = 38)	*P *value	*P#*
Pneumonia	74 (57.8)	23 (67.6)	51 (54.3)	0.195	29 (55.8)	10 (71.4)	19 (50)	0.168	0.802
Respiratory failure	42 (32.8)	13 (38.2)	29 (30.9)	0.432	12 (23.1)	4 (28.6)	8 (21.1)	0.842	0.196
Pulmonary hemorrhage	3 (2.3)	1 (2.9)	2 (2.1)	1.000	1 (1.9)	1 (7.1)	0 (0)	0.269	1.000
Bloody stool	31 (24.2)	16 (47.1)	15 (16.0)	< 0.001	17 (32.7)	7 (50)	10 (26.3)	0.200	0.244
Metabolic acidosis	26 (20.3)	10 (29.4)	16 (17.0)	0.124	10 (19.2)	3 (21.4)	7 (18.4)	1.000	0.869
Hemolysis	61 (47.7)	14 (41.2)	47 (50)	0.377	22 (42.3)	5 (35.7)	17 (44.7)	0.559	0.514
Septicemia	52 (40.6)	15 (44.1)	37 (39.4)	0.628	29 (55.8)	7 (50)	22 (57.9)	0.611	0.064
Anemia	65 (50.8)	18 (52.9)	47 (50)	0.769	28 (53.8)	10 (71.4)	18 (47.4)	0.123	0.709
Electrolyte disturbance	62 (48.4)	21 (61.8)	41 (43.6)	0.070	26 (50)	8 (57.1)	18 (47.4)	0.532	0.849
Peritonitis	30 (23.4)	14 (41.2)	16 (17.0)	0.004	11 (21.2)	7 (50.0)	4 (10.5)	0.002	0.741
Shock	24 (18.8)	8 (23.5)	16 (17.0)	0.405	10 (19.2)	4 (28.6)	6 (15.8)	0.522	0.940
Thrombocytopenia	22 (17.2)	14 (41.2)	8 (8.5)	<0.001	7 (13.5)	5 (35.7)	2 (5.3)	0.004	0.538
Hypoglycemia	17 (13.3)	6 (17.6)	11 (11.7)	0.381	6 (11.5)	2 (14.3)	4 (10.5)	1.000	0.751
Cholestasis	9 (7.0)	1 (2.9)	8 (8.5)	0.486	3 (5.8)	1 (7.1)	2 (5.3)	1.000	1.000
Hypoalbuminemia	36 (28.1)	18 (52.9)	18 (19.1)	<0.001	10 (19.2)	5 (35.7)	5 (13.2)	0.152	0.215
Invasive ventilation	15 (11.7)	5 (14.7)	10 (10.6)	0.748	5 (9.6)	4 (28.6)	1 (2.6)	0.022	0.684
Non-invasive ventilation	22 (17.2)	6 (17.6)	16 (17.0)	0.934	13 (25)	4 (28.6)	9 (23.7)	1.000	0.230
Breath stimulants	20 (15.6)	6 (17.6)	14 (14.9)	0.705	6 (11.5)	2 (14.3)	4 (10.5)	1.000	0.480
Blood transfusion	15 (11.7)	9 (26.5)	6 (6.4)	0.002	7 (13.5)	4 (28.6)	3 (7.9)	0.139	0.746
Apply antibiotics	33 (25.8)	9 (26.5)	24 (25.5)	0.915	15 (28.8)	5 (35.7)	10 (26.3)	0.750	0.000
WBC	11.2 (7.9, 16.4)	13.8 (9.7, 20.6)	10.4 (7.6, 15.5)	0.016	10.5 (7.2, 15.2)	14.1 (5.8, 17.4)	10.3 (7.4, 13.8)	0.470	0.456
WBC ≥ 15×109/L	40 (31.3)	15 (44.1)	25 (26.6)	0.059	14 (26.9)	7 (50.0)	7 (18.4)	0.023	0.566
ANC	6.6 (4.2, 10.1)	8.6 (5.6, 14.3)	6.1 (4.1, 9.2)	0.009	6.9 (4.1, 10.8)	8.5 (4.0, 13.4)	6.5 (4.1, 9.7)	0.369	0.897
ANC ≥ 10× 109/L	33 (25.8)	16 (47.1)	17 (18.1)	0.001	15 (28.8)	6 (42.9)	9 (23.7)	0.313	0.673
LYM	2.3 (1.5, 3.6)	2.0 (1.2, 3.9)	2.4 (1.5, 3.6)	0.478	2.1 (1.2, 3.0)	2.1 (1.0, 2.9)	2.1 (1.3, 3.1)	0.781	0.170
MO	1.2 (0.8, 2.1)	1.4 (0.7, 2.5)	1.1 (0.8, 1.7)	0.234	1.2 (0.7, 1.6)	1.4 (0.7, 2.7)	1.2 (0.8, 1.6)	0.789	0.704
HB	144.1 ± 33.1	141.5 ± 35.0	145.0 ± 32.6	0.593	144.5 ± 32.9	131.2 ± 25.1	149.3 ± 34.3	0.077	0.946
RDW	16.1 (15.2, 17.1)	16.2 (15.5, 17.0)	16.0 (15.1, 17.1)	0.686	16.1 (15.2, 17.2)	16.3 (15.3, 18.4)	16.1 (15.1, 17.1)	0.509	0.781
ALT	10 (8, 17)	11 (8, 19)	10 (7, 17)	0.912	11 (7.3, 17.3)	13 (10, 27)	10 (7, 15)	0.073	0.891
AST	41 (27, 60)	49 (27, 64)	37 (27, 60)	0.167	43 (30, 72)	74 (28, 117)	40 (29, 55)	0.078	0.662
AKP	206 (141, 294)	192 (117, 247)	215 (146, 302)	0.104	173 (137, 260)	189 (140, 354)	167 (134, 249)	0.375	0.385
TBIL	132.2 (100.8, 186.9)	115.8 (99.6, 180.8)	138 (102, 189)	0.366	141.1 (90.7, 199.4)	147.3 (72.4, 241.3)	141 (97, 198)	0.845	0.418
CK	199 (109, 342)	220 (158, 347)	186 (101, 342)	0.358	237 (112, 451)	230 (129, 597)	241 (106, 426)	0.726	0.467
CK-MB	36 (24, 63)	48 (27, 79)	33 (23, 54)	0.031	50 (25, 100)	96 (49.5, 148)	37 (23, 68)	0.016	0.100
CK-MB ≥ 50U/L	40 (31.3)	15 (44.1)	25 (26.6)	0.059	26 (50)	11 (78.6)	15 (39.5)	0.012	0.018
LDH	453 (327, 569)	494 (371, 588)	428 (323, 568)	0.146	510 (371, 701)	631 (431, 813)	475 (319, 599)	0.018	0.200
PAB	72 (50, 88)	71 (49, 86)	73 (51, 95)	0.335	65 (49, 84)	63 (44, 83)	65 (50, 85)	0.592	0.402
Cr	41 (30, 51)	43 (34, 54)	38 (28, 51)	0.182	48 (37, 62)	48 (42, 67)	47.5 (36.0, 60.8)	0.433	0.013
PT	15.5 (129, 17.8)	15.7 (13.9, 17.8)	15.5 (12.8, 17.8)	0.432	14.6 (12.9, 17.8)	17.7 (14.5, 20.0)	13.9 (12.5, 15.9)	0.009	0.428
APTT	47.2 (39.2, 60.9)	55.5 (42.4, 67.0)	45.5 (38.3, 56.2)	0.039	52.5 (43.6, 60.5)	57.1 (48.3, 61.3)	47.3 (42.6, 60.6)	0.216	0.253
≥ 50s	58 (45.3)	22 (64.7)	36 (38.3)	0.011	27 (51.9)	10 (71.4)	17 (44.7)	0.087	0.421

#Comparison between the training and validation cohort variables

The normal range of test indicators: WBC (4-10×109/L), ANC (2.5-7.5×109/L), CK-MB (< 25 U/L), APTT (31 - 40 s)

*ANC* Absolute neutrophil count, *LYM* Lymphocyte absolute value, *MO *Monocyte absolute value, *RDW *Red blood cell distribution width, *HB* Haemoglobin, *ALT *Alanine Aminotransferase, *AST *Aspartate aminotransferase, *AKP* Alkaline phosphatase, *TBIL *Total bilirubin, *CK* Creatine kinase,* CK-MB c*reatine kinase isoenzyme,* LDH *Lactate dehydrogenase, *PAB *Prealbumin, *Cr *Creatinine, *PT *Prothrombin time, *APTT *Activated partial thromboplastin time

We also detected differences of the means of several laboratory indicators between the perforation and non-perforation groups, including elevated WBC (13.8 × 10^9^/L *vs.* 10.4 × 10^9^/L, *P* = 0.016), ANC (8.6 × 10^9^/L *vs.* 6.1 × 10^9^/L, *P* = 0.001), and CK-MB (48U/L *vs.* 33U/L, *P* = 0.031), prolonged APTT (55.5 s *vs.* 45.5 s, *P* = 0.039) in the perforation group. However, there was no statistical difference between the groups of LYM, MO, HB, RDW, ALT, AST, TBIL, AKP, CK, LDH, PAB, Cr, and PT (*P* > 0.05). The validation cohort was also divided into perforation and non-perforation groups, some characteristics were similar to the training cohort, for example, CK-MB (96 U/L *vs.* 37 U/L, *P* = 0.016) in the perforation group was significantly higher than that in the non-perforation group. Comparisons between the training and validation cohorts suggested that only small differences were showed between the demographics, symptoms, complications, and laboratory results (Tables 1 and 2).

### Risk factors for intestinal perforation in NEC

**Fig. 2:**
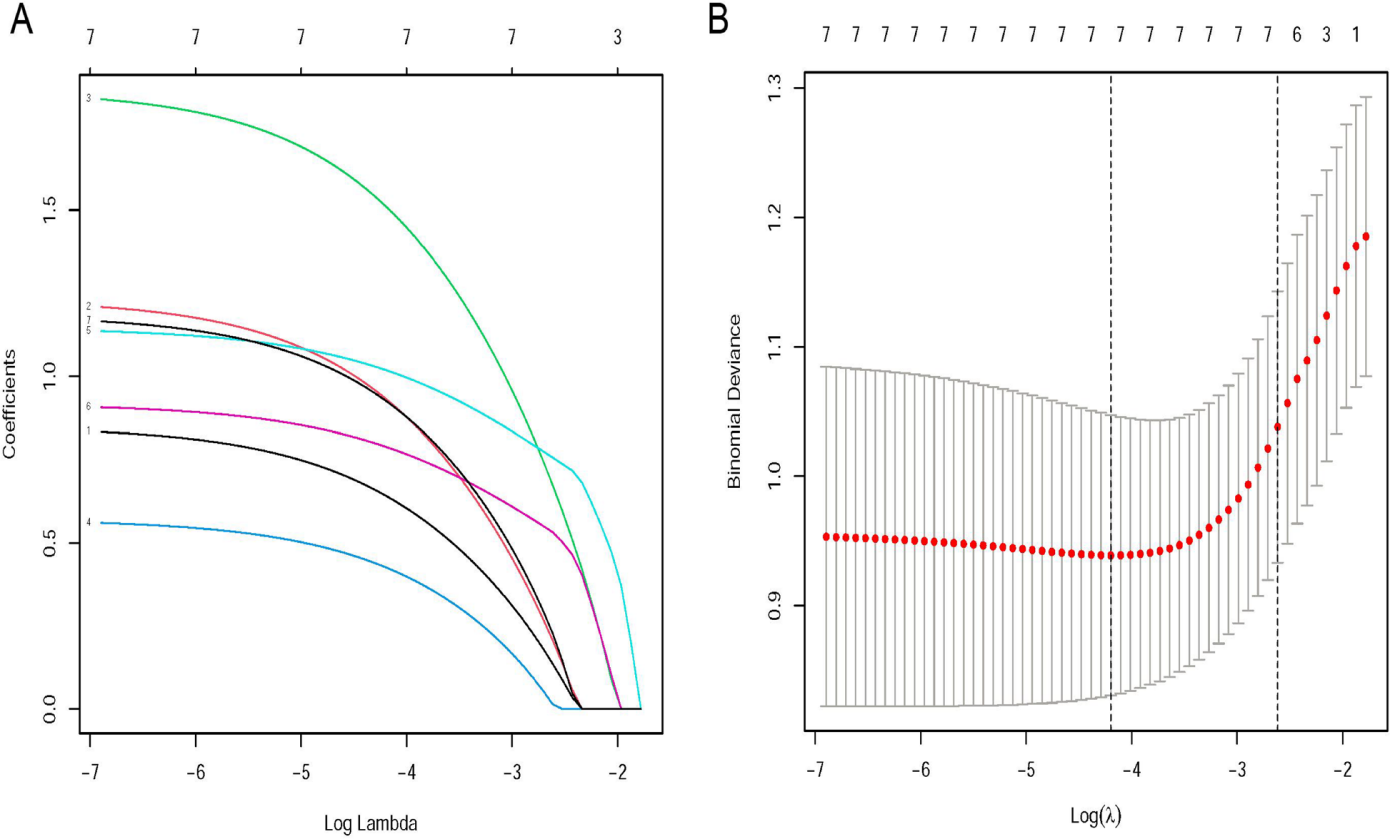
LASSO binary logistic regression model for the selection of clinical indicators. **A** The LASSO model was validated five times using minimum criteria to determine the best parameter (lambda). **B** LASSO coefficient profiles for the seven features were plotted against log(lambda) sequences

Next, we performed univariate and multivariate logistic analysis to identify risk factors for intestinal perforation in neonate with NEC. There were 48 patients with NEC complicated by intestinal perforation confirmed by imaging and operation. By using univariate analysis and LASSO regression of the cohort, we found bloody stool, peritonitis, thrombocytopenia, hypoproteinemia, blood transfusion, ANC ≥ 10 × 10^9^/L, and APTT ≥ 50 s may be risk factors for the occurrence of intestinal perforation in NEC (*P* < 0.05) (Fig. 2). After using multivariate logistic regression analysis, we identified that the presence of bloody stool (OR = 5.60, 95% CI: 1.92, 16.33), thrombocytopenia (OR = 4.74, 95% CI: 1.52, 14.81), hypoproteinemia (OR = 5.56, 95% CI: 1.95, 15.82), and APTT ≥ 50 s (OR = 3.22, 95% CI: 1.21, 8.61), were independent risk factors for the development of intestinal perforation (*P* < 0.05) (Table 3).

**Table 3:** Risk factors related to perforationin in the training cohort

Characteristics	Univariable OR (95% CI)	*P *value	Multivariable OR (95% CI)	*P *value
Weight (g)	－	－	－	－
>1500	1 (ref)	－	－	－
≤ 1500	1.36 (0.57, 3.28)	0.489	－	－
Bloody stool	4.68 (1.96, 11.18)	0.001	5.60 (1.92, 16.33)	0.002
Peritonitis	3.41 (1.43, 8.14)	0.006	－	－
Thrombocytopenia	7.53 (2.78, 20.37)	<0.001	4.74 (1.52, 14.81)	0.007
Hypoalbuminemia	4.75 (2.04, 11.08)	<0.001	5.56 (1.95, 15.82)	0.001
ANC	－	－	－	－
< 10×109/L	1 (ref)	－	－	－
≤ 10×109/L	4.03 (1.71, 9.46)	0.001	－	－
APTT	－	－	－	－
< 50s	1 (ref)	－	－	－
≥ 50s	2.95 (1.31, 6.69)	0.009	3.22 (1.21, 8.61)	0.019
Blood transfusion	5.28 (1.72, 16.26)	0.004	－	－

*ANC* Absolute neutrophil count

### Construction and validation of the nomogram model

**Fig. 3:**
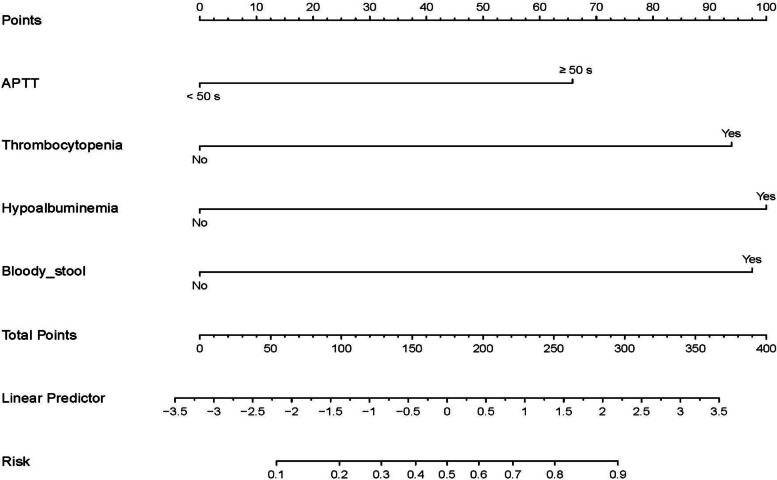
Nomogram model of NEC complicated with intestinal perforation. A nomogram for NEC with intestinal perforation was developed in the training cohort based on APTT, thrombocytopenia, hypoalbuminemia, and Bloody stool

**Fig. 4:**
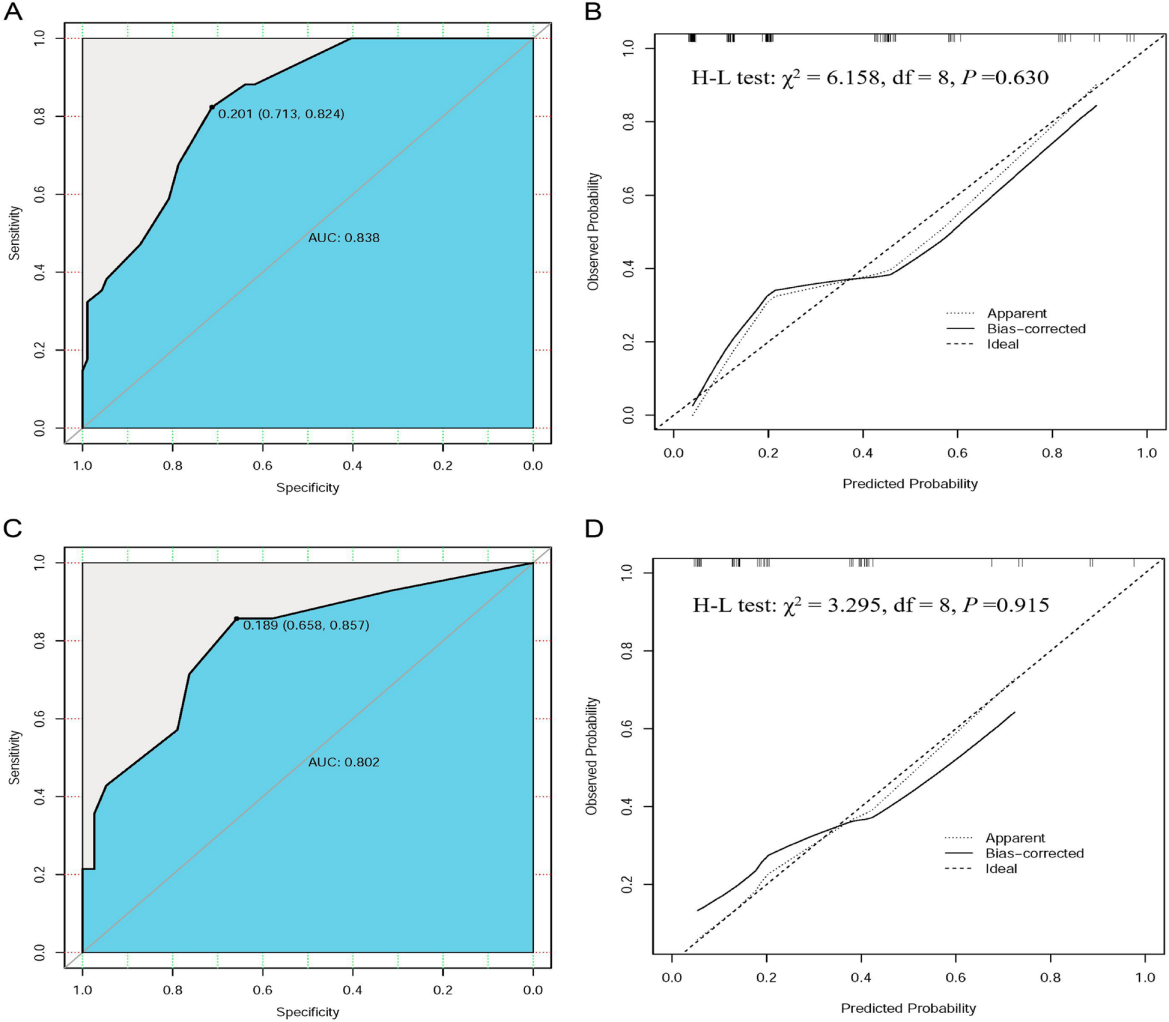
The ROC curves and calibration curves of the nomogram model in training and validation cohorts. **A** Training cohort ROC curve. **B** Validation cohort ROC curve. **C** In the training cohort, the calibration curve for the nomogram mode was showed. **D** In the validation cohort, the calibration curve for the nomogram mode was showed. The dotted line represents the performance of the model. The diagonal line represents the ideal prediction

The independent risk predictors of intestinal perforation in the NEC patients from the training cohort were applied to R software to construct the nomogram model (Fig. 3). Thrombocytopenia and bloody stool brought the highest risk to patients, followed by hypoproteinemia and APTT ≥ 50 s. For each neonate with NEC, a higher score means a higher risk of intestinal perforation. The area under the receiver operating curve (AUC) for the nomogram in the training cohort was AUC = 0.838 (95% CI: 0.768–0.908), C-index = 0.838 (Fig. 4A), and the calibration curve of the model (χ^2^ = 6.158, df = 8, *P* = 0.630) revealed a good agreement between the nomogram predicted probability and the actual observed result of intestinal perforation (Fig. 4B). In addition, the probability of intestinal perforation occurrence in NEC patients from the validation cohort was calculated by the nomogram model. The area under the curve (AUC) for the validation cohort was 0.802 (95% CI: 0.659–0.944) (Fig. 4C), which also had a well-calibrated curve (χ^2^ = 3.295, df = 8, *P* = 0.915) in the assessment of risk (Fig. 4D).

**Fig. 5:**
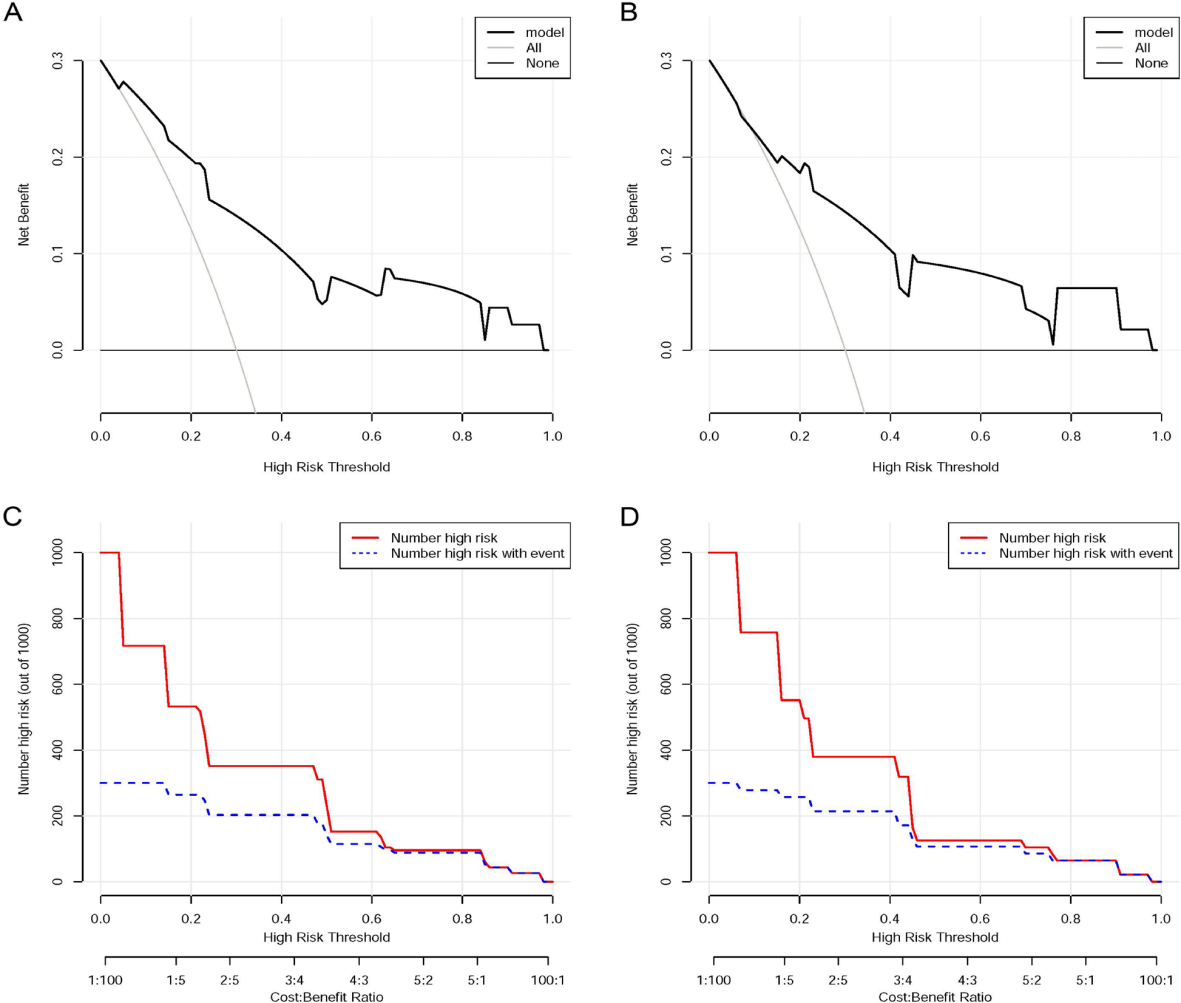
The decision analysis curves and clinical impact curves of the nomogram model in training and validation cohorts. **A** The DCA curve for the model of the training cohort. **B** The DCA curve for the model of the validation cohort. A standardized net risk is showed on the y-axis, while risk thresholds and cost-effectiveness ratios are showed on the x-axis. **C** Clinical impact curve based on risk factor risk models in the training cohort. **D** Clinical impact curve based on risk factor risk models in the validation cohort. Out of 1,000 neonates, in the solid red line, the number of babies considered high risk at each risk threshold is showed, whereas in the blue dashed line, the number of newborns who were considered true positives (cases) is showed

Finally, we used decision curve analysis (DCA) and clinical impact curve (CIC) analysis to evaluate the nomogram model’s potential for clinical application. The decision curves showed that if the threshold probability is higher than 0.05 in the training cohort (0.07 in the validation cohort), the nomogram model had a higher standardized net benefit to predict the probability for neonates with NEC developing intestinal perforation (Fig. 5A and B). The clinical impact curves also showed that the nomogram has high efficiency in the identification of perforated NEC neonates at a relatively higher risk threshold (Fig. 5C and D). All the decision and clinical impact curves indicated that the nomogram might be promising in clinical decision-making.

## Discussion

NEC is the most common necrotizing inflammatory disease of the intestine during the neonatal period, which has a complex etiology and is prone to multiple organ damage, including intestinal perforation at an early stage, posing a severe threat to the health of the neonates [7, 26]. Therefore, early identification of NEC combined with intestinal perforation and active intervention is one of the key factors in treating this disease. In this study, we first summarized the characteristics of neonatal necrotizing enterocolitis (NEC) complicated by intestinal perforation or not. Then we performed univariate, lasso regression screening and multi-factor logistic regression analysis on the clinical, complications, and laboratory findings of neonates with NEC. The results revealed that bloody stool, thrombocytopenia, hypoproteinemia, and APTT ≥ 50 s were independent risk factors for intestinal perforation in neonates with NEC. Based on these risk factors, we established a nomogram model to predict the occurrence of intestinal perforation in neonates with NEC. Our study can help clinically understand the risk factors for intestinal perforation in neonates with NEC, which can be used for early intervention in clinical practice for neonates with a high risk of intestinal perforation to improve the survival of patients with NEC.

Previous studies showed that the smaller the gestational age and birth weight, the higher the incidence of NEC [23, 27]. But in our study, among the 180 confirmed NEC cases, most patients had a relatively large gestational age and heavier birth weight. In their follow-up NEC study, Lin et al. reported that 77.8% of NEC patients were late preterm or term infants, and 90.3% of the infants had a birth weight of more than 1500 g based on a 10 years retrospective study [28]. Another study involving 598 NEC patients showed that the gestational age and birth weight of NEC perforated group were 36.57 (33.43–38.86) weeks and 2500 (2020–3200) g, respectively, and the gestational age and birth weight of NEC non-perforated group were 37.86 (35.14–39.21) weeks and 2800 (2250–3250) g, respectively [29]. In addition, a multicenter study that eventually included 449 NEC patients showed that 238 premature infants (53.0%) and 211 full-term infants (47.0%) had intestinal perforation, importantly, 47 full-term infants also had intestinal perforation [30]. These results and our study revealed that we need to note that the risk of NEC in late preterm and term infants may be increasing.

Pathological hematochezia frequently indicates gastrointestinal disorders such as neonatal necrotizing small bowel colitis, congenital megacolon, or systemic coagulation disorders [31, 32]. NEC is the most common cause of bloody stool. It is associated with complications such as severe electrolyte disturbances, intestinal strictures, and intestinal perforation. The average time to onset of NEC in newborns with positive occult blood tests was 7 days, according to a study of fecal occult blood tests [33]. An international survey of pediatricians from 26 countries revealed that bloody stool, abdominal cramps, low platelet counts, and elevated lactate levels increased the incidence of NEC in 82%, 72%, 56%, and 45% of respondents, respectively [34]. Our study found that the presence of bloody stool is a high-risk factor for intestinal perforation in neonates with NEC, and may contribute approximately 98 points to the nomogram model score. The development of NEC and its intestinal complications must therefore be warned of in neonates who present with bloody stool early in clinical practice.

Thrombocytopenia is a common clinical manifestation of NEC, typically appearing within 72 h of the onset of NEC [35], and its level is directly proportional to the severity and prognosis of NEC, previous studies indicate that platelets were significantly reduced in NEC neonates with Bell stage III [36]. In this study, we found that thrombocytopenia is an independent risk factor for intestinal perforation in neonates with NEC. And thrombocytopenia increased the nomogram model score by approximately 93 points. Maheshwari established a mouse model of NEC and confirmed that the tissue factor released by thrombin and macrophages promotes platelet depletion [37]. It has also been suggested that thrombocytopenia may be associated with the severity of the intestinal injury, with platelet counts below 100 × 10^9^/L being linked to the development of complications such as intestinal necrosis [26]. It has also been showed that transfusion of blood products accelerates intestinal pathologies [38], We also found a higher proportion of patients who were given blood transfusions in the perforation group than that in the non-perforation group, which was identified as a potential risk factor for the occurrence of intestinal perforation by univariate analysis but was not statistically significant when multiple factors were considered. On the contrary, it has also been suggested that blood product transfusion may have a protective effect on the intestine [23], so whether a transfusion is associated with intestinal perforation in NEC needs to be proven in large data sets.

Hypoalbuminemia, defined as a serum albumin concentration of less than 30 g/L, is usually due to increased bleeding or intestinal loss in patients with NEC. It is also because infants are susceptible to compromise albumin levels in disease states due to their poor ability to synthesize albumin. Our study has showed that hypoalbuminemia was an independent risk factor for the development of intestinal perforation in NEC, and combined hypoalbuminemia increased the nomogram model score by 100 points. At the same time, there are studies that have showed that hypoalbuminemia is associated with poor prognosis in NEC, including increased complications and reduced patient survival [39, 40]. A previous study demonstrated that hypoalbuminemia was a potent, dose-dependent independent predictor of poor prognosis in NEC, with every 10 g/L decreases in serum albumin concentration associated with a 137% increase in mortality, 89% increase in morbidity, and 28% and 71% increases in intensive care unit and hospitalization time, respectively, and that complication rates may be reduced when serum albumin levels are raised above 30 g/L [41]. It has also been noted that an increase or decrease in serum albumin levels is a valuable indicator of disease recovery or deterioration [42]. Therefore, the occurrence of hypoalbuminemia in neonates with NEC in clinical practice needs to be alerted to the development of intestinal perforation, and timely treatment of the primary diseases, albumin infusion, and other interventions for hypoalbuminemia may improve the prognosis of the neonates with NEC.

Patients with NEC commonly exhibit abnormal coagulation. It has been demonstrated that the expression of genes related to coagulation and anticoagulation is significantly altered in patients with NEC, and that these abnormalities lead to a procoagulant state, which causes altered intestinal vascular permeability and impaired microcirculation, resulting in progressive deterioration of intestinal lesions [43], including mesenteric thrombosis, intestinal ischemia, and intestinal perforation as complications [44, 45]. Our results revealed that APTT ≥ 50 s is an independent risk factor for intestinal perforation in NEC and could increase the nomogram model score by about 66 points. An increased APTT is associated with a high incidence of surgery and a poor prognosis for neonates with NEC [46]. Consequently, early dynamic monitoring of blood coagulation status and correct the coagulation function in time may help reduce complications in neonates with NEC.

## Conclusion

In summary, our study has established a visual predictive model scoring system with good discrimination and accuracy based on the presence of bloody stool, thrombocytopenia (PLT < 100 × 10^9^/L), hypoproteinemia (< 30 g/L), and APTT ≥ 50 s, which are independent risk factors for intestinal perforation in neonates with NEC, and may serve as an essential basis for clinical guidance and application. However, the limitations of this study include: firstly, it is a single center, retrospective case–control study with a small sample size, which may lead to a certain degree of selection bias. Secondly, due to the long time span of sample sources in this study, limited early diagnosis and treatment levels, and limited understanding of the disease, some potential risk factor data that may affect the occurrence of NEC have not been fully preserved, such as prenatal ultrasound examination, preeclampsia, chorioamnionitis, etc. Therefore, further research through multicenter and prospective trials is needed.

## Data Availability

The datasets used and/or analysed during the current study available from the corresponding author on reasonable request.
